# Increasing adherence and collecting symptom-specific biometric signals in remote monitoring of heart failure patients: a randomized controlled trial

**DOI:** 10.1093/jamia/ocae221

**Published:** 2024-08-22

**Authors:** Sukanya Mohapatra, Mirna Issa, Vedrana Ivezic, Rose Doherty, Stephanie Marks, Esther Lan, Shawn Chen, Keith Rozett, Lauren Cullen, Wren Reynolds, Rose Rocchio, Gregg C Fonarow, Michael K Ong, William F Speier, Corey W Arnold

**Affiliations:** Department of Molecular, Cell, and Developmental Biology, University of California, Los Angeles, Los Angeles, CA 90024, United States; Department of Ecology and Evolutionary Biology, University of California, Los Angeles, Los Angeles, CA 90024, United States; Department of Radiology, University of California, Los Angeles, Los Angeles, CA 90024, United States; Department of Radiology, University of California, Los Angeles, Los Angeles, CA 90024, United States; Department of Radiology, University of California, Los Angeles, Los Angeles, CA 90024, United States; Department of Medicine, University of California, Los Angeles, Los Angeles, CA 90024, United States; Department of Radiology, University of California, Los Angeles, Los Angeles, CA 90024, United States; Office of Advanced Research Computing, University of California, Los Angeles, Los Angeles, CA 90024, United States; Office of Advanced Research Computing, University of California, Los Angeles, Los Angeles, CA 90024, United States; Office of Advanced Research Computing, University of California, Los Angeles, Los Angeles, CA 90024, United States; Office of Advanced Research Computing, University of California, Los Angeles, Los Angeles, CA 90024, United States; Department of Medicine, University of California, Los Angeles, Los Angeles, CA 90024, United States; Department of Medicine, University of California, Los Angeles, Los Angeles, CA 90024, United States; Department of Radiology, University of California, Los Angeles, Los Angeles, CA 90024, United States; Department of Bioengineering, University of California, Los Angeles, Los Angeles, CA 90024, United States; Department of Radiology, University of California, Los Angeles, Los Angeles, CA 90024, United States; Department of Bioengineering, University of California, Los Angeles, Los Angeles, CA 90024, United States; Department of Pathology & Laboratory Medicine, University of California, Los Angeles, Los Angeles, CA 90024, United States

**Keywords:** mHealth, heart failure, remote monitoring

## Abstract

**Objectives:**

Mobile health (mHealth) regimens can improve health through the continuous monitoring of biometric parameters paired with appropriate interventions. However, adherence to monitoring tends to decay over time. Our randomized controlled trial sought to determine: (1) if a mobile app with gamification and financial incentives significantly increases adherence to mHealth monitoring in a population of heart failure patients; and (2) if activity data correlate with disease-specific symptoms.

**Materials and Methods:**

We recruited individuals with heart failure into a prospective 180-day monitoring study with 3 arms. All 3 arms included monitoring with a connected weight scale and an activity tracker. The second arm included an additional mobile app with gamification, and the third arm included the mobile app and a financial incentive awarded based on adherence to mobile monitoring.

**Results:**

We recruited 111 heart failure patients into the study. We found that the arm including the financial incentive led to significantly higher adherence to activity tracker (95% vs 72.2%, *P* = .01) and weight (87.5% vs 69.4%, *P* = .002) monitoring compared to the arm that included the monitoring devices alone. Furthermore, we found a significant correlation between daily steps and daily symptom severity.

**Discussion and Conclusion:**

Our findings indicate that mobile apps with added engagement features can be useful tools for improving adherence over time and may thus increase the impact of mHealth-driven interventions. Additionally, activity tracker data can provide passive monitoring of disease burden that may be used to predict future events.

## Introduction

Heart failure (HF) is a severe health condition associated with high morbidity, mortality, and health resource use.^1^ In the United States, 6.2 million adults have HF, and roughly $31 billion is spent annually on HF-related costs, the majority of which (75%-80%) is attributed to hospitalizations.^1^^,^^2^ With approximately 1 million annual hospitalizations, HF stands as a leading cause of hospital admissions in people aged over 65.^3^ Approximately 1 in 4 HF patients are readmitted within 30 days of discharge, and 1 in 2 patients are readmitted within 6 months of discharge.^4^ A total of 400 000 death certificates in 2018 cited HF as a contributing factor, and it is predicted that by 2030, cases will surge by 25%.^2^^,^^4^^,^^5^ The cumulative impact of this chronic disease includes a substantial economic burden, with projected costs reaching up to $69.7 billion by 2030, a 127% growth since 2012.^2^

Clinical guidelines suggest that adherence to self-care recommendations is vital to a favorable HF prognosis,^6–8^ and nearly half of all HF readmissions are preventable with enhanced adherence to self-care behavior after discharge.^9–11^ It is clear from the evidence highlighted above that identifying effective adherence-enhancing tools and methods is essential to promote positive HF health outcomes. Despite these recommendations, the ability of HF patients to engage in adequate self-care is frequently compromised by various factors, such as old age, comorbidities, multiple symptoms, and a lack of available support.^12^ This compromise contributes to the persistent challenge of poor adherence to self-care recommendations among HF patients.^13^^,^^14^

Mobile health (mHealth) interventions have been expanding to diverse populations^15^ and may be a preferable and less intensive method to deliver medical care.^16^^,^^17^ By including the use of mHealth technologies in the home monitoring of HF, patients may be more inclined to play an active role in lifestyle modifications that are intended to improve their health outcomes and prevent rehospitalization.^18^ The use of mobile applications can supplement mHealth interventions by providing patients with an avenue to monitor their progress and receive adherence notifications.^19^ Present-day home monitoring interventions employ wireless sensors, telephone services, websites, and home visits from nurses.^20–22^ Several studies using mHealth in HF patients have suffered from small sample size and poor patient adherence to monitoring.^22–26^ For instance, 1 trial found that only 55.4% of patients randomized to telemonitoring used the given technology-based intervention at least half of the time at month 1, and 51.7% at month 6.^24^ The reported low adherence levels and inconclusive results associated with such studies are in part attributable to the high monitoring burden of home interventions. In investigating strategies to overcome these barriers, the combined use of gamification and financial incentives has yet to be extensively employed in mHealth adherence studies.^27^

The primary outcome of this study was to evaluate differences in adherence levels to 3 mHealth home monitoring regimens in HF patients involving a combination of devices, a mobile app, and financial incentives. Our hypothesis was that including a mobile app would boost adherence to using study devices. Secondarily, we hypothesized that activity tracker data would correlate with reported HF symptoms. Our results provide new insights into tailoring mHealth interventions to drive adherence and suggest the possibility of passively monitoring HF symptoms using activity trackers.

## Methods

### Study population

This prospective study included patients with HF at the University of California, Los Angeles (UCLA) medical center. The study was approved by the UCLA Institutional Review Board (IRB) and was funded under a grant from the National Heart, Lung, and Blood Institute (R01HL141773). Patients with an HF diagnosis were identified using our institution’s electronic health record (EHR). English-speaking adults aged 50-80 who had a diagnosis of HF and owned a smartphone were eligible to participate in the study. Exclusion criteria included participants who were receiving hemodialysis, had received an organ transplant or were on an organ transplant waiting list, or did not have the cognitive or physical ability to participate.

### Subject recruitment

Initial contact with individuals meeting eligibility criteria was established through email and text messages to assess their interest in participating. Subsequently, research personnel engaged with those who responded positively to the initial outreach via phone conversations, providing additional information about the study and conducting the verbal consent process for those expressing interest. To ensure an interested individual was capable of using their smartphone, they first participated in a daily survey delivered via text messages for 1 week. The survey consisted of 3 questions: (1) how much did symptoms of heart failure limit your life yesterday? (2) were you able to take your medication as prescribed yesterday? and (3) did you follow your recommended diet yesterday? Participants who completed at least 5 of the 7 surveys were invited to participate in the full study for 180 days, a monitoring period that captures disease dynamics and has been used in previous studies of telemonitoring for HF patients that report adherence measures.^23^^,^^25^

### Study groups

To investigate the impact of different mHealth regimens on adherence, each participant was independently randomized into one of the 3 groups. The 3 groups used 3 different combinations of an activity tracker, a scale, a mobile app, and a financial incentive. The groups are defined as follows: devices only (Group D), devices and mobile app (Group D + A), and devices and mobile app with a financial incentive (Group D + A + F). Group D received only the devices (ie, the activity tracker and scale). Group D + A received the devices as well as a smartphone application developed by the study team. Group D + A + F received the devices, the study smartphone application, and a financial incentive based on the participant’s adherence, which maxed out at $150 and was paid at the completion of the monitoring period. Group D serves as the control group in our study, enabling a comparison between participants using only the devices and those using the devices in conjunction with a complementary mobile app (Group D + A), as well as those with the additional incentive of a financial reward integrated into the app (Group D + A + F). Although only Group D + A + F received a direct financial incentive for completing the study, all participants were allowed to retain the devices after the study period concluded, regardless of their adherence level. Over the phone, all participants were taught how to use the devices, and those in Groups D + A and D + A + F were also taught how to access the smartphone application and navigate all features within the application. Groups were also measured using standard surveys (described below). A prior study of coronary heart disease patients that used an app to increase medication adherence observed an effect size (ES) of 0.6.^28^ Using this ES for our study, a target sample size of 36 subjects per group results in a power of 0.80.

### Study devices and mobile app

#### Fitbit Charge 4 and Fitbit Charge 5

The Fitbit Charge 4 (FC4), released in 2020, or the Fitbit Charge 5 (FC5), released in 2021, were used in all groups to provide daily feedback on a variety of parameters, such as physical activity. The FC4 was replaced with the FC5 due to Fitbit’s product release cycle and all groups had equivalent proportions of both devices. Both devices are equipped with a standard array of sensors, including a 3-axial accelerometer, an altimeter, an optical heart-rate tracker, a vibration motor, and a GPS receiver. Identical data points were collected from both models. The devices track, record, and deliver real-time information on step count, heart-rate, sleep, and active minutes. Subjects were directed to wear their Fitbit on their wrists at all times, except when charging the device. Subjects were also instructed on how to use the Fitbit smartphone application. Battery life for both the FC4 and FC5 is 3-5 days, with a charging time of approximately 1 hour.

#### BodyTrace scale

Participants were provided with a BodyTrace scale (BodyTrace Inc., New York, NY, United States) for daily weighing. The BodyTrace scale includes a cellular modem with a factory-installed SIM card. The scale arrives ready for use with 4 AA batteries and requires no user configuration. When a participant uses the scale to weigh themselves, the weight data is automatically synced and transmitted to a cloud-based database through the cellular modem.

#### myHeartCare mobile application

**Figure 1.**
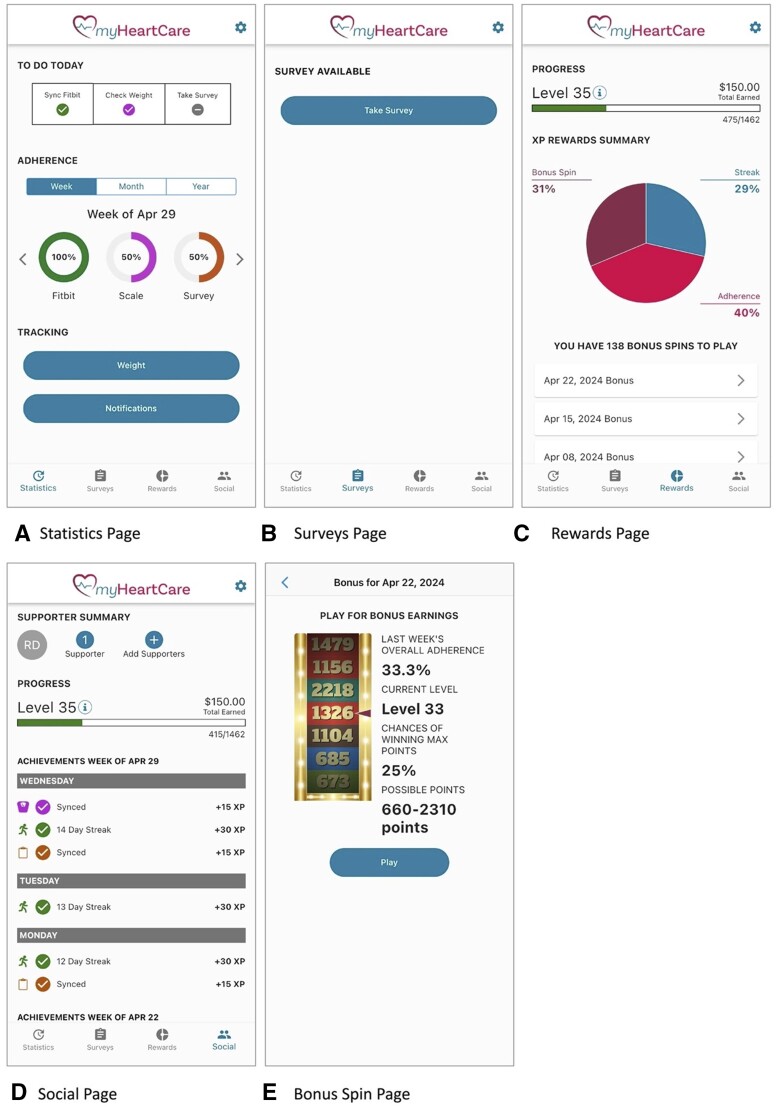
Selected screenshots of the myHeartCare user interface: (A) statistics page, (B) surveys page, (C) rewards page, (D) social page, and (E) bonus spin page.

For this study, the research team developed *myHeartCare*, a cross-platform (iOS or Android) mobile app capable of linking to Fitbit trackers and BodyTrace scales. The app was designed by the study team, which included input from physicians, nurses, and coordinators who interacted with HF patients. performed, who wore Fitbits and weighed themselves over several weeks to ensure the technical usability and accuracy of the app. Groups D + A and D + A + F, the mobile app users, downloaded this app and were instructed on navigating its features. The app is structured into 4 sections: (1) adherence statistics, (2) surveys, (3) rewards, and (4) social (Figure 1). Within the statistics page, users are presented with a daily “To-Do” list, prompting them to sync their Fitbit to their smartphone, weigh themselves, and complete the daily survey. The gamification process is initiated through notifications and reminders, with participants receiving a daily push notification at 9 am to sync their Fitbit, weigh themselves, and take the daily survey. Points serve as incentives for adherence, with points accruing and displaying on the rewards page upon completing each daily activity and with extra points awarded when a participant completes all tasks multiple days in a row. Additionally, participants have the opportunity for a weekly “Bonus Spin,” in which they are randomly assigned bonus points within a range that is determined based on how adherent they were during the week. Users can track their progress through a leveling system represented by a progress bar on the Rewards page, “leveling up” as points are accumulated. Each level requires more points than the previous level to achieve. Finally, social support mechanisms are integrated into the app via the social page, allowing users to invite friends and family to view their adherence history and send encouraging messages.

Points in myHeartCare act as internal rewards for Group D + A participants but translate to up to $150 in earnings for Group D + A + F participants. The amount received is based on a subject’s level at the end of the monitoring period. Each level awards $5 with the maximum level possible capped at 30.

### Baseline survey

Those who consented to participate in the study were administered a baseline survey consisting of questions relating to demographic information and 2 institutional review board-approved questionnaires—the Minnesota Living with Heart Failure Questionnaire (MLHFQ)^29^ and Patient-Reported Outcomes Measurement Information System (PROMIS) Global Health.^30^ These questionnaires were used to measure the HF patients’ health-related quality of life through patient-reported outcomes using a 5-point Likert scale. Following this survey, patients were officially consented to the study and randomized to one of the 3 study groups.

### Follow-up survey

After 180 days of monitoring, participants were contacted over the phone and administered a follow-up survey. The survey consisted of the same 2 questionnaires administered in the baseline survey—the MLHFQ and PROMIS Global Health. In addition to these questionnaires, participants were asked to rate their experiences with the Fitbit and the BodyTrace scale. The survey assessed factors of these devices, such as helpfulness, ease of incorporation into daily life, and whether each device helped the participant adhere to their care plan. Participants were then offered an option for an additional 180 days of Fitbit and BodyTrace scale data collection, which did not require completion of the daily survey. Seventy-eight participants agreed to the additional data collection.

### Data collection and analysis

The study team implemented connections to vendor APIs that were used to pull participant data and update the myHeartCare app in real-time when a task had been completed for subjects in the D + A and D + A + F groups. The server also received the responses to the daily questionnaire for the D + A and D + A + F groups. Three metrics were used to define adherence: (1) syncing the Fitbit, (2) checking weight, and (3) completing the daily survey. Adherence is reported in 2 ways: (1) as the percentage of subjects who were adherent more than 50% of the time, and (2) as a continuous value, computed as the fraction of days the task was completed (eg, Fitbit synced) divided by the total number of days in the time period of analysis (eg, 30 days). Kruskal-Wallis tests were employed to assess differences in adherence levels among the 3 groups for weight checking and Fitbit syncing adherence. Post-hoc pairwise comparisons were performed using the Mann-Whitney U test with Benjamini-Hochberg correction. Survey adherence between the 2 app groups was compared using the Mann-Whitney *U* test with Benjamini-Hochberg correction. These nonparametric tests were chosen due to the non-normal distribution of our data, which was confirmed by the Kolmogorov-Smirnov test. Additionally, adherence to the 3 tasks was compared among participants across ejection fraction (EF) and New York Heart Association (NYHA) class within all 3 intervention groups.

Heart failure symptom severity was assessed in the D + A and D + A + F groups using the question “Yesterday, how much did symptoms of heart failure limit your life?” The 3 possible question responses were “not really” (0), “moderately” (1), and “extremely” (2). The correlation between daily step count and symptoms was evaluated using the analysis of variance (ANOVA) test across the 3 HF symptom severity levels and the paired sample *t*-test for pairwise comparisons.

Free-text responses from the follow-up survey were analyzed using an inductive thematic approach. Verbatim transcripts of survey answers were reviewed, and emerging themes were identified and coded. Subsequently, comments were categorized into distinct thematic codes. Upon naming the themes, illustrative quotations from transcripts were selected for each theme.

## Results

**Figure 2.**
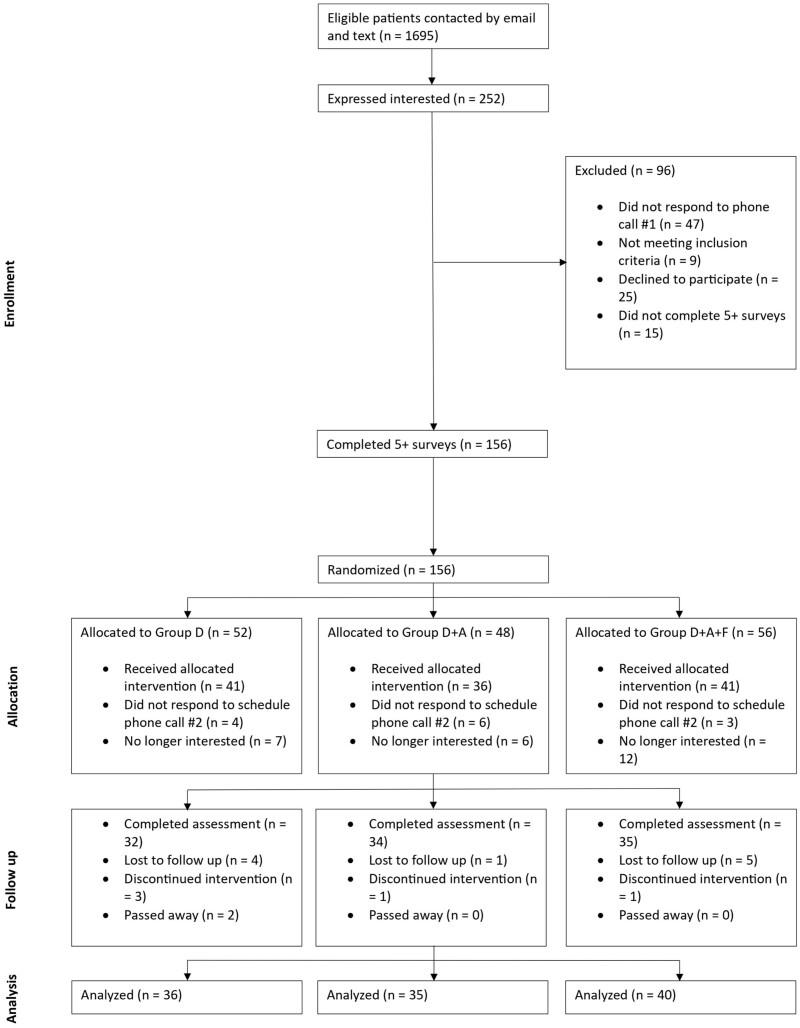
Consort diagram presenting the progression of patients in the study.

**Figure 3.**
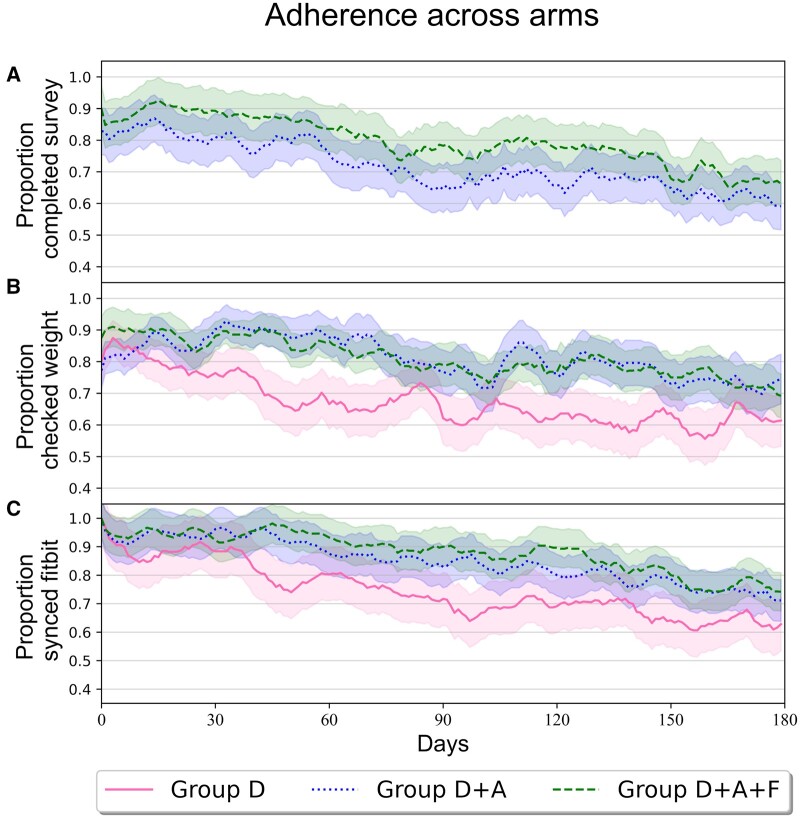
Plots of adherence rates for the 3 primary tasks completed by study participants, stratified by intervention group. Group D is represented by a solid pink line, Group D + A is represented by a dotted blue line, and Group D + A + F is represented by a dotted green line. Shaded regions represent standard errors. Moving average smoothing with a window of 7 days was used for each group.

**Figure 4.**
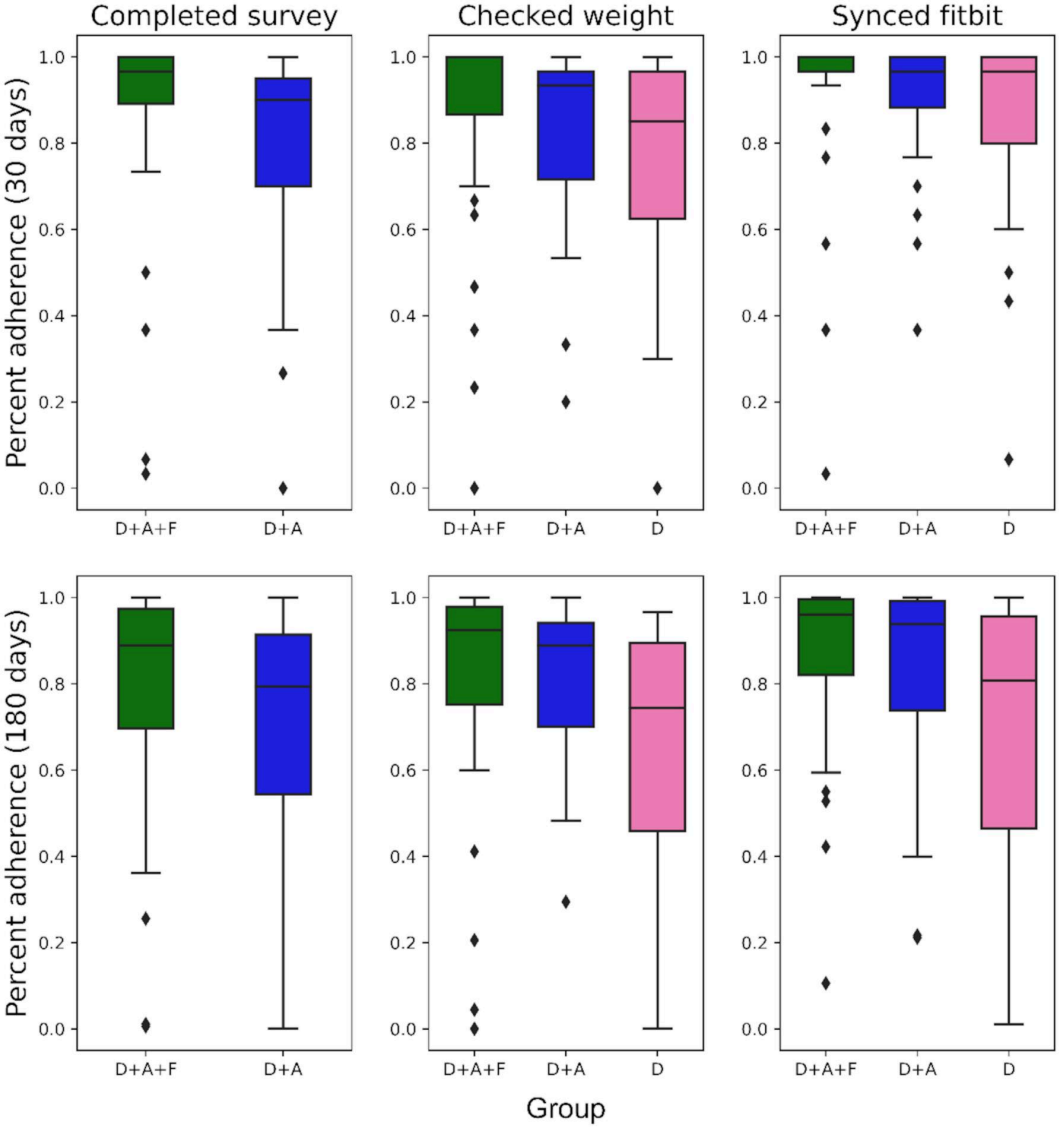
Box plots of device adherence and mobile app survey completion (D + A and D + A + F groups only) for the different groups at 30 and 180 days. Unit of analysis is a subject’s fraction of days the task was completed divided by the time period. Two subjects did not have cellular service at their home, which prevented the weight scale from syncing and thus resulted in 0% adherence for the duration of the study.

Figure 2 presents the consort diagram detailing the trial’s progression. In total, 1695 patients identified through UCLA’s EHR received email or text communications to assess their interest in participating in the study. Of these, 252 (14.9%) gave a positive response and were contacted by phone to verify eligibility and obtain consent; 96 (38.1%) individuals were excluded from the study. Among these exclusions, 81 either failed to respond to the phone call, were ineligible, or declined participation. The remaining 15 patients, although consenting to the surveys, did not complete the required 5 out of 7 daily surveys necessary for continued involvement A total of 156 patients (61.9%) successfully met the daily survey requirements and were randomly assigned to one of 3 groups: Group D with 52 patients, Group D + A with 48 patients, and Group D + A + F with 56 patients. Across these groups, 13 participants did not respond to the study, and 25 said they were no longer interested, leaving 118 participants who enrolled and received the intervention. These 118 participants represent 46.8% of those who initially expressed interest in the study and 7% of those who were initially contacted by email and text. Out of the 118 participants, 10 were lost to follow-up, 5 discontinued the intervention, and 2 expired. The final analysis included both those who completed the assessment and those who were lost to follow-up, totaling 111 patients. In total, 111 participants completed the 180 study between July 2021 and April 2023. Group D had 36 patients, while Group D + A and Group D + A + F had 35 and 40 patients, respectively. The demographics of the participants who completed the study are shown in Table 1. Our results demonstrate that participants within groups D + A and D + A + F had a higher adherence rate over time for the 3 individual measured tasks compared to Group D. Moreover, there was a greater decline in Group D’s adherence rates relative to the groups which utilized the mobile app as their participation in the study progressed. Table 2 displays the average adherence of each group to the 3 measured tasks at 1, 30, 90, and 180 days. Figure 3 displays this information for each day of participation in the study and Figure 4 shows box plots of subject adherence over 30-day and 180-day periods.

**Table 1.** Demographics of the 111 participants who completed the study, organized by category and intervention group.

Category	Sub-category	Group D (36)	Group D + A (35)	Group D + A + F (40)
Gender	Male	26 (72.22%)	29 (82.86%)	27 (67.50%)
	Female	10 (27.78%)	6 (17.14%)	13 (32.50%)
	Non-binary/third gender	0	0	0
	Prefer not to say	0	0	0
Race	White	27 (75.00%)	27 (77.14%)	27 (67.5%)
	Black/African American	4 (11.11%)	2 (5.71%)	5 (12.50)
	Asian	3 (8.33%)	4 (11.43%)	2 (5.00%)
	American Indian/Alaskan	0	0	1 (2.50%)
	Native Hawaiian/Pacific Islander	0	0	0
	More than one race	1 (2.78%)	0	4 (10.00%)
	Don’t know/refuse	1 (2.78%)	2 (5.71%)	1 (2.50%)
Age	50-60	10 (27.78%)	10 (28.57%)	14 (35.00%)
	61-70	17 (47.22%)	12 (34.29%)	12 (30.00%)
	71-80	9 (25.00%)	13 (37.14%)	14 (35.00%)
Education	High school	3 (8.33%)	0	3 (7.50%)
	Some college/associate degree/trade school	8 (22.22%)	6 (17.14%)	13 (32.50%)
	Bachelor’s degree	7 (19.44%)	15 (42.86%)	3 (7.50%)
	Master’s degree or above	18 (50.00%)	14 (40.00%)	21 (52.50%)
	Don’t know/refuse	0	0	0
Income	$0-$25 000	2 (5.56%)	3 (8.57%)	3 (7.50%)
	$25 001-$50 000	5 (13.89%)	1 (2.86%)	6 (15.00%)
	$50 001-$75 000	1 (2.78%)	2 (5.71%)	4 (10.00%)
	$75 001 or more	26 (72.22%)	26 (74.29%)	25 (62.50%)
	Don’t know/refuse	1 (2.78%)	3 (8.57%)	2 (5.00%)
Hispanic/Latino	No	33 (91.67%)	33 (94.29%)	35 (87.50%)
	Yes	3 (8.33%)	2 (5.71%)	5 (12.50%)
	Don’t know/refuse	0	0	0
Etiology	Ischemic	18 (50.00%)	21 (60.00%)	22 (55.00%)
	Non-ischemic	17 (47.22%)	12 (34.29%)	15 (37.50%)
	Do not have	1 (2.78%)	2 (5.71%)	3 (7.50%)
LVEF median	25-34	6 (16.67%)	8 (22.86%)	10 (25.00%)
	35-44	8 (22.22%)	7 (20.00%)	7 (17.50%)
	45-54	8 (22.22%)	4 (11.43%)	7 (17.50%)
	55-64	7 (19.44%)	5 (14.29%)	4 (10.00%)
	65-75	2 (5.56%)	4 (11.43%)	1 (2.50%)
	Do not have	3 (8.33%)	5 (14.29%)	7 (17.50%)
	<25	2 (5.56%)	2 (5.71%)	4 (10.00%)
NYHA	1	2 (5.56%)	5 (14.29%)	5 (12.50%)
	2	16 (44.44%)	18 (51.43%)	10 (25.00%)
	3	4 (11.11%)	4 (11.43%)	12 (30.00%)
	4	0	0	2 (5.00%)
	1-2	2 (5.56%)	0	1 (2.50%)
	2-3	3 (8.33%)	4 (11.43%)	3 (7.50%)
	3-4	1 (2.78%)	0	2 (5.00%)
	Do not have	8 (22.22%)	3 (8.57%)	6 (15.00%)

**Table 2.** Device adherence and mobile app survey completion (D + A and D + A + F groups only) for the different groups at 1, 30, 90, and 180 days.

	Group	Day 1	Day 30	Day 90	Day 180
**Proportion checked weight**	D	0.800	0.7571	0.629	0.571
	D + A	0.771	0.971	0.743	0.765
	D + A + F	0.875	0.923	0.775	0.700
**Proportion synced Fitbit**	D	1.00	0.857	0.743	0.600
	D + A	1.00	1.00	0.886	0.735
	D + A + F	1.00	0.897	0.850	0.775
**Proportion completed survey**	D + A	0.829	0.882	0.657	0.559
	D + A + F	0.900	0.897	0.775	0.650

### Fitbit syncing

Regarding Fitbit syncing, adherence to greater than 50% of days was documented in 97.5% of Group D + A + F participants after the first 30 days in the study and in 95% at the end of the 180-day period. In Group D + A, 100% of participants met the >50% adherence threshold after the first 30 days, and 91.4% at the end of the study. For Group D, 97.2% of participants met this threshold after the first 30 days, and 72.2% at the end of the study.

Continuous adherence values (Table 2) are defined as the percentage of people in a group that were adherent for each day. When comparing continuous adherence values, a significant difference in adherence (*P* <.05) was found between Group D and Group D + A + F during the first 30 (*P* = .008, ES = 0.33), 60 (*P* = .003, ES = 0.39), 90 (*P* = .005, ES = 0.37), 120 (*P* = .008, ES = 0.35), 150 (*P* = .006, ES = 0.37), and 180 (*P* = .01, ES = 0.35) days of the study.

### Self-weighing

For self-weighing, adherence to greater than 50% of days was achieved by 92.5% of Group D + A + F participants after the first 30 days, and 87.5% at the end of the study. In Group D + A, 94.3% of participants met the adherence threshold both after the first 30 days and at the 180-day mark. For Group D, 94.4% of participants achieved this after 30 days, and 69.4% at the 180-day period of the study ending.

Statistical analysis of the continuous adherence values revealed a significant difference between Group D and Group D + A + F during the first 30 (*P*<.001, ES = 0.46), 60 (*P*<.001, ES = 0.53), 90 (*P*<.001, ES = 0.51), 120 (*P*<.001, ES = 0.46), 150 (*P*<.001, ES = 0.44), and 180 (*P* = .002, ES = 0.42) days of the study. There was a notable difference between Group D + A and Group D + A + F for the first 60 (*P* = .043, ES = 0.27, not significant under Benjamini-Hochberg correction) days of the study. A significant difference was found between Group D and Group D + A for the first 60 (*P* = .016, ES = 0.33), 90 (*P* = .013, ES = 0.34), 120 (*P* = .009, ES = 0.36), 150 (*P* = .014, ES = 0.34), and 180 (*P* = .019, ES = 0.32) days of the study.

### Survey taking

Participants in Group D did not have the option of taking the daily survey as they did not have the mobile app. For Group D + A + F, 95% were adherent for over 50% of days at 30 days, and 87.5% at 180 days. For Group D + A, 88.6% of participants met this threshold after the first 30 days, and 82.9% at the end of the study.

Statistical analysis indicated a notable difference between the continuous adherence values for Group D + A and Group D + A + F for the first 30 (*P* = .0329, ES = 0.28, not significant under Benjamini-Hochberg correction) and 60 (*P* = .0385, ES = 0.28, not significant under Benjamini-Hochberg correction) days of the study.

### Intention-to-treat analysis

Of the 5 subjects who withdrew from the study, 2 were in Group D (withdrew at week 4 and week 16), 1 was in Group D + A (withdrew at week 12), and 2 were in Group D + A + F (withdrew at week 2 and week 6). We performed an intention-to-treat analysis that included these individuals by setting their adherence to zero for the days following their study withdrawal. The inclusion of these days resulted in a new significant difference in continuous adherence values between Group D and Group D + A for Fitbit syncing at 60 (*P* = .04, ES = 0.27), 90 (*P* = .03, ES = 0.30), 120 (*P* = .03, ES = 0.30), 150 (*P* = .03, ES = 0.29), and 180 (*P* = .04, ES = 0.28) days of the study.

### Fitbit syncing, self-weighing, and survey taking across EF and NYHA class

Ejection fraction measures the percentage of blood the left ventricle pumps out with each contraction and the NYHA functional classification is a system to classify the severity of HF and a patient’s functional ability. Overall, no significant differences in adherence levels to the 3 tasks for all 3 intervention groups stratified by EF (<50% vs >50%) and NYHA class (1-2 vs 3-4) were found with the exception of a higher rate of Fitbit syncing among participants in Group D with NYHA class 3-4 compared to those with NYHA class 1-2.

### Correlation between HF symptoms and activity

Symptoms of HF responses were recorded only for participants who had the option to take the daily survey (D + A and D + A + F groups). Fewer daily steps were found to be correlated with increased symptoms of HF for Group D + A and Group D + A + F. A significant difference in steps was found between those responding “not really” and “extremely” (*P* = .001), and “moderately” and “extremely” (*P* = .005). There was no significant difference in steps on days in which participants responded “not really” or “moderately.” No participants reported difficulty using their devices due to their HF symptoms.

### Follow-up survey feedback

Participants across all 3 groups found the study devices helpful and provided primarily positive feedback on the Fitbit and scale, as seen in Tables 3–5. Table 3 shows that over 50% of study participants found the Fitbit and scale either “very helpful” or “extremely helpful.”

**Table 3.** Participant evaluation of Fitbit and scale by intervention group.

	Group D, *N* = 32		Group D + A, *N* = 34		Group D + A + F, *N* = 35	
Survey questions and available responses	*n*	%	*n*	%	*n*	%
**On a scale of 1-5, with 1 being not at all helpful and 5 being extremely helpful, how helpful did you find the Fitbit?**						
1—Not at all helpful	3	9.4	1	2.9	1	2.9
2—Slightly helpful	7	21.9	4	11.8	6	17.1
3—Moderately helpful	6	18.8	5	14.7	5	14.3
4—Very helpful	8	25.0	11	32.4	9	25.7
5—Extremely helpful	8	25.0	13	38.2	14	40.0
**On a scale of 1-5, with 1 being not at all helpful and 5 being extremely helpful, how helpful did you find the scale?**						
1—Not at all helpful	2	6.3	3	8.8	1	2.9
2—Slightly helpful	1	3.1	1	2.9	4	11.4
3—Moderately helpful	9	28.1	6	17.6	3	8.6
4—Very helpful	9	28.1	8	23.5	6	17.1
5—Extremely helpful	11	34.4	16	47.1	21	60.0

**Table 4.** Free responses to open-ended questions regarding Fitbit and corresponding themes.

Themes and testimonials
**Increased awareness of one’s health condition and activity levels**
“The Fitbit…made me conscious of my condition. It was a physical reminder.” (Group D + A, Male, 60)
“I liked the curiosity factor, I paid attention to the number of steps each day.” (Group D + A + F, Male, 70)
“It [the Fitbit] is easily available as a constant reminder.” (Group D + A, Male, 80)
**Increased motivation to improve activity levels and maintain an active lifestyle**
“I liked the get-up-and-walk notification so I would get my walking in.” (Group D + A, Male, 60)
“The Fitbit…inspired me to walk at least 1 mile a day. I tried to get 7,000-8,000 steps per day.” (Group D + A, Male, 75)
“The Fitbit was reinforcing, getting [giving] praise for doing different activities.” (Group D + A + F, Female, 72)
“It [the Fitbit] provided a vehicle for me to monitor my physical activity and set goals for myself, as I am very goal oriented. I could set goals and make sure I achieve them. It motivated me to exercise more than I would before.” (Group D + A + F, Male, 58)
“It [the Fitbit] motivated me to walk more, check my calories, and reach goals I set for myself. It also got my family interested in my goals.” (Group D + A + F, Male, 66)
“I liked that the Fitbit counts steps… I am already trying to beat my record.” (Group D, Female, 70).
**Facilitated adherence to care plan with greater awareness and precision**
“I liked that I could check my heart rate during exercise, as my doctor advised me to keep my heart rate at 60 so I would monitor that. I also liked that the Fitbit alerted me to a high heart rate so I could relax and bring it back down.” (Group D + A + F, Female, 62)
“It [the Fitbit] helped me adhere to my care plan because…if I felt my heart struggling, I would watch my heart beats to make sure my heart rate didn’t get too high.” (Group D + A + F, Female, 60)
“It [the Fitbit]…reminded me to follow instructions my doctor gave me.” (Group D + A + F, Female, 71)
“I liked that it [the Fitbit] kept track of my heart rate, since I have had ventricular tachycardia (VTAC).” (Group D + A + F, Male, 78)
“It [the Fitbit] helps me keep track of my fluid intake.” (Group D, Male, 69)
“I could correlate my steps to my fatigue.” (Group D + A + F, Male, 54)
**Challenges and difficulties in using the device**
“I had trouble figuring out how to use the device, so I was unable to use its full capability.” (Group D + A + F, Male, 61)
“It was difficult to navigate the screens, [the] appearance is unattractive.” (Group D + A + F, Male, 65)
“It was mildly easy to incorporate [the Fitbit] into my daily life. It is geared towards a younger generation.” (Group D + A + F, Male, 54)
“A minor drawback was that it took some time to figure out to use it [the Fitbit] and get in the habit of wearing it.” (Group D + A + F, Male, 61)
“For my age, it [the Fitbit] was too technologically advanced.” (Group D + A + F, Male, 78)
“Working the device was difficult and got in my way. It was hard to set up and the buttons were not easy to use. Flipping between screens was difficult, I was hoping it would be easier to use.” (Group D, Male, 69)

**Table 5.** Free responses to open-ended questions regarding scale and corresponding themes.

Theme and testimonials
**Increased connectivity of scale and app (for Groups D + A and D + A + F)**
“I liked that it got me into the habit of weighing myself every day (something I hated doing before). I liked that it connected straight through to the phone.” (Group D + A + F, Female, 78)
“I liked that the weight could be tracked over time.” (Group D + A + F, Male, 76)
“I liked that it prevented me from having to manually log my weight everyday.” (Group D + A, Male, 56)
“I liked that the scale transmitted my weight to the app so I can track it, so I can be mindful of my weight.” (Group D + A, Male, 60)
**Increased motivation to track weight and fluid intake**
“Since I started, I was able to lose quite a bit of weight (started at 266 lbs and ended at 250 lbs).” (Group D + A, Male, 78)
“My weight fluctuates easily if I don’t eat so it was a reminder for me to stay at a certain number and not let my weight drop too much.” (Group D, Male, 52)
“The scale helped me adhere to my care plan because I could track my water retention and adjust medications as necessary.” (Group D, Male, 76)
“It helped me adhere to my care plan since I was able to be more mindful of what I ate.” (Group D + A, Female, 51)
“The scale helped me adhere to my care plan because knowing my weight is useful to me because I am borderline diabetic.” (Group D + A + F, Male, 78)

## Discussion and conclusion

The study’s overall enrollment rate of 7% and sample size of 111 participants align with comparable figures in other mHealth interventions for HF. For example, a telemonitoring study for HF reported that 4.7% of initially assessed patients were randomized and received the allocated intervention.^25^ Another study found that 2.8% of assessed patients received the intervention, yielding a sample size of 184 participants.^31^ Furthermore, the remote recruitment processes we put in place due to the COVID-19 pandemic are more scalable than previous in-person approaches that require study coordinators to meet subjects in clinics. The low overall recruitment rate is primarily due to patients who did not respond to our initial communication via text or email (1443 out of 1695 patients contacted). Among the 252 individuals who responded, 68% enrolled in the daily surveys and 91% of these individuals completed at least 5 out of the 7 surveys, suggesting that the technological demands associated with study enrollment were manageable for the majority of interested participants. While the sample size is modest and the requirement for survey completion for enrollment may have encouraged a more engaged participant pool, statistically significant differences in adherence levels between groups were observed.

Our results demonstrate that incorporating a mobile app and financial incentives significantly increases adherence to monitoring over time, and in the context of an mHealth intervention, even modest increases in adherence could translate to better health outcomes. In addition, subjects generally had positive experiences using the devices and the mobile app. In other clinical trials with cardiovascular disease participants that utilized remote interventions integrating gamification and financial incentives, these interventions were also found to promote physical activity and positive outcomes.^32^ A review of gamification and incentives in mHealth apps found that studies generally reported improved or sustained optimal medication adherence outcomes and positive health behaviors, further supporting our results.^27^

Intriguingly, we found a significant correlation between activity and disease-specific symptoms, providing evidence that an activity-based intervention could target underlying disease rather than signal artifacts. This finding suggests the potential of employing activity trackers as a low-burden, indicative approach for monitoring symptoms of HF. Such use could play a role in forecasting clinical decline among HF patients—recognizing shifts in the severity of their condition as possibly reflected in activity data could prompt patient care teams to intervene or reconsider treatment strategies as necessary.

Despite higher adherence relative to previous work, we still observed adherence decay over time. Future modifications to our study protocol could incorporate providing an incentive at regular time intervals to sustain long-term adherence, such as device upgrades and more frequent financial reward distributions rather than waiting until the end of the study. However, while this approach may help keep participants engaged throughout the study, it is also possible that a larger financial reward at the end of the study period could be more motivating. Another challenge we observed was anecdotal response data that indicated some subjects struggled with incorporating the monitoring technology into their daily lives. Identifying these individuals early during recruitment and providing them with additional support may mitigate this issue.

Limitations of this study include the requirement of smartphone ownership and the higher socioeconomic status (SES) of many participants. A review of HF and SES found that lower SES was associated with increased incidence of HF, greater hospital readmission rates, and lower survival.^33^ The higher SES of study participants can be attributed to the geographical area of recruitment, which was the surrounding area near UCLA, which is not representative of the entire United States.^34^^,^^35^ Furthermore, HF incidence varies across race and ethnicity but is also more prevalent among older adults.

While technical literacy was not an inclusion criterion for the study, another limitation is the potential generalizability to older populations, given the correlation between technical literacy and age, and the fact that HF is more prevalent with increasing age.^36^ Additionally, our study duration of 180 days cannot assess the longer-term durability of adherence. Finally, despite our use of randomization to control for confounders across arms, there may be population biases, in addition to those mentioned above, that could limit the generalization of our results.

Looking to the future, given that the app, activity tracker, and scale send data in real time, there is an opportunity for population monitoring using machine learning approaches to identify patients at risk of HF exacerbation. Our results, which show a significant correlation between daily step counts and HF symptoms, indicate that there may be a predictive signal in activity data. Our ongoing work includes gathering more data and integrating diverse sensor signals and app responses into sequential machine learning techniques, with the goal of forecasting different HF outcomes.

## Data Availability

The data underlying this article will be shared on reasonable request to the corresponding author.
